# Effects of Print Publication Lag in Dual Format Journals on Scientometric Indicators

**DOI:** 10.1371/journal.pone.0059877

**Published:** 2013-04-03

**Authors:** Petr Heneberg

**Affiliations:** Third Faculty of Medicine, Charles University in Prague, Prague, Czech Republic; University of Illinois-Chicago, United States of America

## Abstract

**Background:**

Publication lag between manuscript submission and its final publication is considered as an important factor affecting the decision to submit, the timeliness of presented data, and the scientometric measures of the particular journal. Dual-format peer-reviewed journals (publishing both print and online editions of their content) adopted a broadly accepted strategy to shorten the publication lag: to publish the accepted manuscripts online ahead of their print editions, which may follow days, but also years later. Effects of this widespread habit on the immediacy index (average number of times an article is cited in the year it is published) calculation were never analyzed.

**Methodology/Principal Findings:**

Scopus database (which contains nearly up-to-date documents in press, but does not reveal citations by these documents until they are finalized) was searched for the journals with the highest total counts of articles in press, or highest counts of articles in press appearing online in 2010–2011. Number of citations received by the articles in press available online was found to be nearly equal to citations received within the year when the document was assigned to a journal issue. Thus, online publication of in press articles affects severely the calculation of immediacy index of their source titles, and disadvantages online-only and print-only journals when evaluating them according to the immediacy index and probably also according to the impact factor and similar measures.

**Conclusions/Significance:**

Caution should be taken when evaluating dual-format journals supporting long publication lag. Further research should answer the question, on whether the immediacy index should be replaced by an indicator based on the date of first publication (online or in print, whichever comes first) to eliminate the problems analyzed in this report. Information value of immediacy index is further questioned by very high ratio of authors’ self-citations among the citation window used for its calculation.

## Introduction

Time spent between manuscript submission and their final publication may greatly vary among different journals, journal publishers, and may affect, e.g., the decision to which journal to submit a manuscript [1]. The publication lags are comparable between those published in the open access mode and under the “reader-pays” model, while the manuscript submitted to journals published or endorsed by the learned societies or other professional organizations are available to their readers following much longer time lag [2].

To overcome the problem of significant time lag between manuscript submission (or acceptance) and its publication, several strategies appeared. In 1978, the Stanford Linear Accelerator Center Preprint/Antipreprint list debuted, consisting of a database set up on an IBM mainframe with separate fields for year, biweekly period, institutional abbreviation, preprint number, author, title, citation, and publication status [3]. Several similar preprint archives appeared through 1980s and 1990s, receiving even a mention in *Science*, where physicist Paul Ginsparg, founder of the Los Alamos archive, was cited as condemning the emergence of new preprint servers as “Balkanization”, complaining that a scientist would need to submit his or her preprints to multiple distinct servers and browse them every morning for new submissions [4]. In 2001, the librarian Cecelia Brown noticed that the documents published in arXiv.org only receive citation rate similar to the print journals, while the examined three dozens of prominent physics and astronomy journals remained cited equally as before the arXiv.org inception in 1991 [5]. In 2007, Henk F. Moed provided the evidence that inclusion of a paper in arXiv.org accelerates its citations (probably due to the fact that arXiv.org makes papers available earlier) but does not affect its total citation counts over [6]. Although the self-archiving probably did not reach its full possibilities, another major player entered the scene in 1990s and 2000s – online availability of archive, current, and in press papers at the web sites of the journal publishers.

Online availability decreased the both the circulation and readership of print journal copies by more than one half [7]. Severe print use decline occurred across a broad range of subject fields, including, e.g., astronomy, biology, computer science, engineering or geology. Only certain fields were reported to be affected less, with top-tier general science journals affected least (15% decrease) [7]. Numerous studies shown at the turn of the Millennium that faculty and students prefer online materials to print [8]–[10]. The ease of access, increasing functionality and broadening back file coverage of online materials allow more time-efficient work, instant access to a more diverse material, and thus higher productivity.

Since most of the online sources contain also the articles in press, these documents are equally available to the web-preferring faculty and students as are the online editions of articles assigned to their volumes and issues. Use of the in press articles is also supported by their inclusion to Scopus or PubMed, although they are excluded from, e.g., Web of Science. Although John McDonald of Caltech reported increased citation rate following the switch of examined journals from the print to the dual print-online format, he also noticed the increase of citation rates before the switch [7], which questions the results and calls for their corroboration. In food research, online posting was reported to shorten the total publication lag by 29%, while the highest advances were reported for several journals published by Elsevier [11]. In mid 2000s, the average paper was published online nearly three months before its print edition (while there were extensive differences between the major publishers). Interestingly, a significant minor group of journals was also reported to publish their papers online later than in print. The latter behavior was especially prominent in economical journals published by Cambridge University Press [12].

Online publication of any article is known to be responsible for the majority of its citations [13]. In case of conference articles in computer science and related areas, the mere online availability of the articles was reported to be responsible for 157% increase in the mean citation rate [13]. When assessing the articles published in the top-tier journals only, the increase is even more prominent, independently on the year of publication [13]. Interestingly, although the total number of downloads correlates with the total number of citations and with the journals’ impact factor, downloads and citations have very different obsolescence patterns. In a subset of oncology journals, the average cited half-life was reported as 5.6 years, while the mean usage half-life for the same group of journals was only 1.7 years for the year 2006 [14]. Number of citations may also increase when multiple copies of the single article are available at different web sites [15]. Clearly, there are numerous indices suggesting the considerable impact of the online publication of in press articles at the speed of scientific findings’ transmission to the community, and at the associated scientometric indicators.

Per O Seglen [16] claimed that short publication lag supports high journal self-citation rate and is associated with high journal impact factor. This is valid for the print-only or online-only model of publishing. However, when utilizing the model of dual print–online publishing, the highest gains should be obtained under the conditions involving short lag between submission and online publication of in press articles, but long lag between online of in press articles and their allocation to print issues. Here I analyze the influence of delayed allocation of online-available in press articles to print issues. The analysis includes ten journals with the highest total counts of articles in press according to Scopus, three journals with the highest counts of articles in press appearing online in 2010–2011, and *PLoS ONE* as an example of online only journal, which cannot be affected by the above phenomenon.

## Materials and Methods

**Figure 1:**
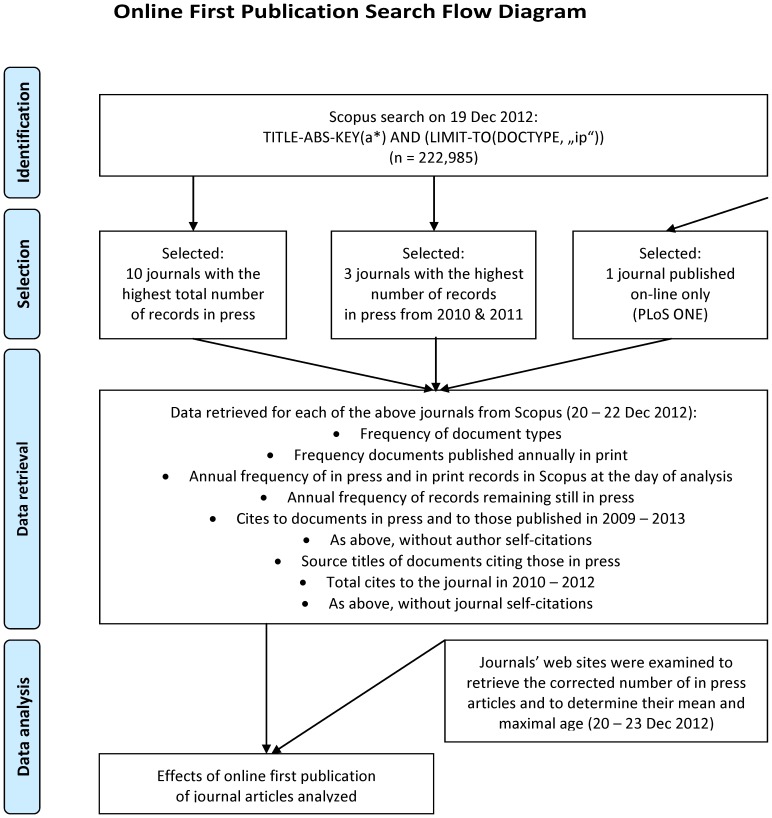
Flowchart of the study on online first publication of journal articles.

The initial search to identify journals with high numbers of in press journal articles was performed using the Elsevier’s Scopus database, which (contrary to the Web of Science) contains relatively well updated records of in press articles. The journals were identified using the search for random articles with words starting with the letter “a” in their title, abstract, or keywords: TITLE-ABS-KEY(a*) AND (LIMIT-TO(DOCTYPE, “ip”)), date search performed: 19 Dec 2012. In press articles are defined as papers that have been accepted for publication and are made available online before they are formally published. These articles do not have any volume or issue number and thus no date of publication in print form [12]. Following the initial search, the above search was restricted to the papers appearing in press in 2010 or 2011 only (Fig. 1).

Since the citation databases, including Scopus, are known to contain relatively high number of errors [17]–[18], the frequency of in press documents and their distribution in time was verified at the journals’ web sites.

Publication lag caused by delayed print publication of online available in press articles was calculated based on the information provided at web sites of all 13 journals analyzed. Number of in press articles contained at the journals’ web sites at the day of analysis was assessed according to the release date for each month between January 2011 and December 2012, and for each year of the preceding period. Cumulative publication lag (*Σ_L_*) of a particular journal was calculated by summarizing the evidence for publication lag (*L_i_*) of all the in press documents analyzed (*Σ_L_ = L_1_+L_2_+…+L_n_*). Mean publication lag (*λ_L_*) of a manuscript was calculated by dividing the cumulative publication lag by the number of in press documents present at the journals’ web sites at the day of analysis (*n*) (λ_L = _ Σ_L_*n^−1^). The date of release of the newest and oldest in press record was recorded for each of the journals. The same was recorded based on the Scopus profile of each particular journal, while part of the earliest in press Scopus records was directly verified for presence of the final prints at the journals’ web sites. When indicated, number of citations to the documents in press was also adjusted to the length of their visibility online by dividing the number of total citations received by the mean publication lag to receive the number of citations per year.

Authors’ self-citations were defined as those when at least one of the authors of a cited document was the same person as one of the authors of the citing document. The authors’ self-citation rate was calculated by dividing the number of self-citations by the total number of all citations received to the evaluated set of documents in a defined period of time. In effect, this rate indicated the probability that any given citation, drawn randomly from the population originally sampled, will be a self-citation [19]. Similarly, journals’ self-citations were defined as those originating from the same source journal title as the cited document. When analyzing the citations retrieved from the Scopus database, it should be noted that Scopus does not display citations from documents being currently in press, although these documents are included in the database. Considering the citations to the documents in press, citations to such documents cannot consist of journals’ self-citations unless the journals’ editor decided to allocate any article appearing later in press to appear earlier in the print issue. The authors’ self-citations were analyzed based on the information received from Scopus as total citation counts of the analyzed documents, and as the citation counts without authors’ self-citations (obtained when applying the command “Exclude from citation overview: Self citations of all authors”). The journals’ self-citations were analyzed based on the information received from Scopus as total citation counts of the analyzed documents, and as the citation counts without journals’ self-citations (obtained when applying the command “Exclude journal self citations” in the Scopus Journal Analyzer). Citations to documents released by the 13 analyzed journals as in press, and to those published by the same journals in print in 2009, 2010, 2011, 2012 and 2013 were collected.

Statistical analyses, including the paired t-test, sign test, and one-way ANOVA with Tukey’s pairwise comparisons were performed in PAST version 2.14 [20].

## Results

### In Press Document Counts

The initial Scopus search identified the following ten journals as having the highest total number of in press articles:

- *International Journal of Cardiology (Int. J. Cardiol*., 1,181 articles in press at a date of search)
- *Journal of Applied Polymer Science (J. Appl. Polym. Sci*., 813 articles in press)
- *Journal of Thermal Analysis and Calorimetry (J. Therm. Anal. Calorim*., 750 articles in press)
- *Journal of Radioanalytical and Nuclear Chemistry (J. Radioanal. Nucl. Chem*., 725 articles in press)
- *International Journal of Advanced Manufacturing Technology (Int. J. Adv. Manuf. Technol*., 685 articles in press)
- *Optik* (645 articles in press)
- *Applied Microbiology and Biotechnology (Appl. Microbiol. Biotechnol*., 608 articles in press)
- *Environmental Monitoring and Assessment (Env. Monit. Assess*., 587 articles in press)
- *Bulletin of Experimental Biology and Medicine (Bull. Exp. Biol. Med*., 571 articles in press)
- *Oncogene* (567 articles in press).

**Figure 2:**
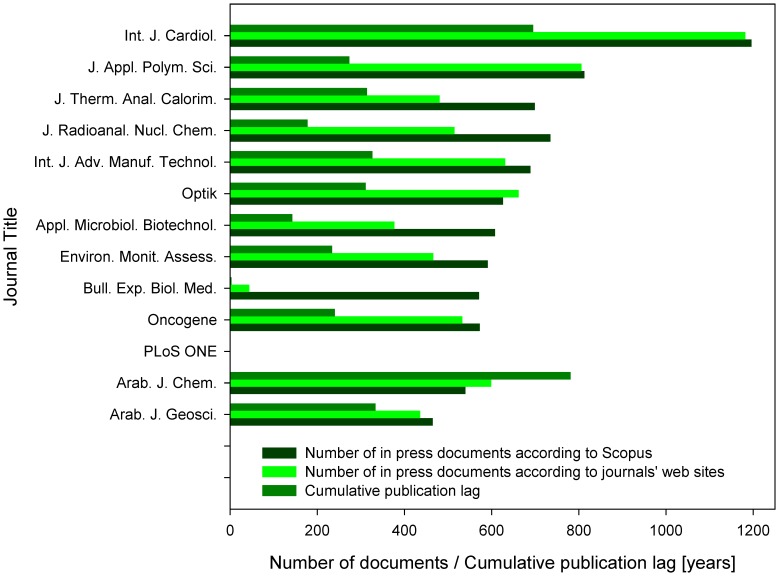
Number of documents in press. Indicated are the numbers of documents in press included in Scopus, numbers of documents in press released at the journals’ web sites, and the cumulative publication lags obtained by summarizing the evidence for publication lag for all the in press documents released at the journals’ web sites (shown in years). Analyses included 13 journal titles representing ten journals with the highest total number of in press articles, three journals with the highest number of in press articles released in 2010 or 2011 and the online-only journal *PLoS ONE*. Cumulative publication lag (*Σ_L_*) of a particular journal was calculated by summarizing the evidence for publication lag (*L_i_*) of all the in press documents analyzed (*Σ_L_ = L_1_+L_2_+…+L_n_*).

**Figure 3:**
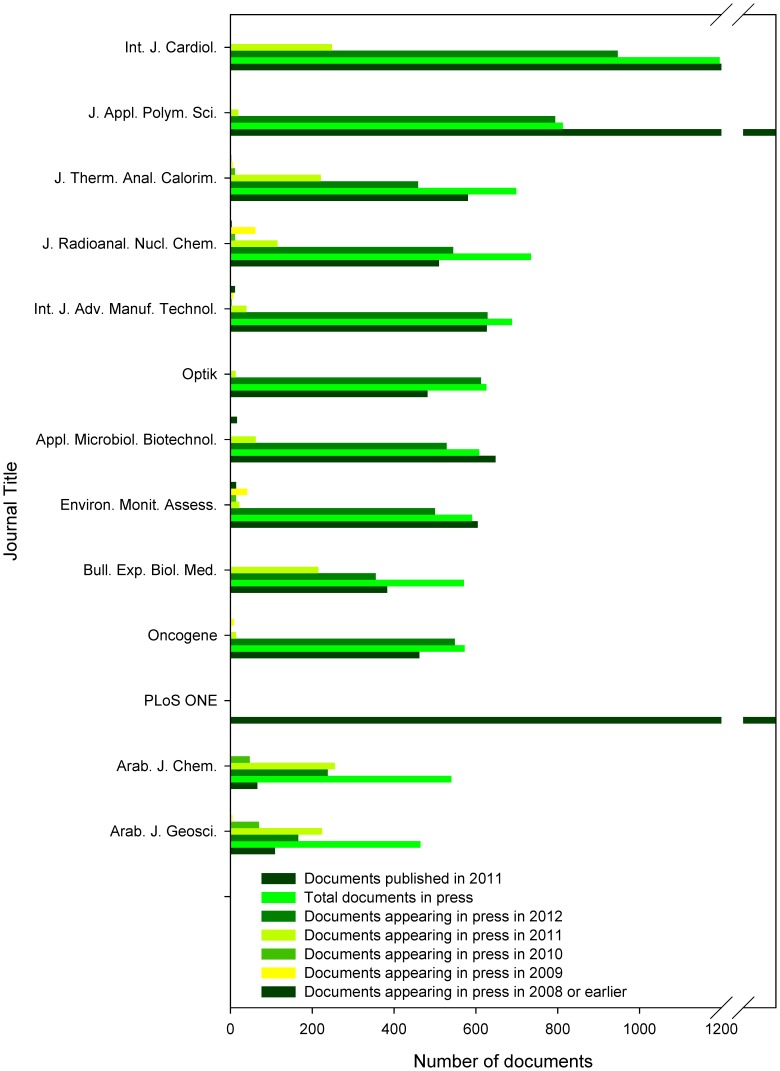
Release dates of documents in press. Indicated are the total numbers of documents published in 2011 (according to Scopus), total numbers of documents in press (according to Scopus), and frequency of release years among documents indicated as in press at the time of analysis (indicated are years 2012, 2011, 2010, 2009, and in 2008 or earlier, all according to Scopus).

In addition, included was also *PLoS ONE* as typical example of an online only journal, where the online publication date represents the final publication date. Besides that, the analyses included also three journals, which contained the highest numbers of articles in press published online in 2010 or 2011: *Arabian Journal of Chemistry* (*Arab. J. Chem.*, 302 articles in press published online in 2010 or 2011, 12^th^ when ranked according to the total number of articles in press), *Arabian Journal of Geosciences* (*Arab. J. Geosci.*, 294 articles in press published online in 2010 or 2011, 20^th^ when ranked according to the total number of articles in press), and the above-listed *J. Therm. Anal. Calorim.* (287 articles in press published online in 2010 or 2011, 3^rd^ in when ranked according to the total number of articles in press) (Fig. 2–3).

Verification of the 20 most cited documents indicated by Scopus as in press at the analysis date revealed that the all 20 of them were already assigned to a journal issue and thus should no longer be listed as in press. Thus, the total numbers of documents in press were retrieved also from the 13 journals’ web sites (Fig. 2) to corroborate the data obtained from Scopus. The numbers retrieved were slightly lower when compared to those obtained from Scopus (mean_web sites_  = 517.9, mean_Scopus_  = 623.5, paired *t*-test *p*<0.05, t  = 2.378, sign test *p*<0.05, r  = 10, n  = 13). While some journals had similar numbers of documents in press contained at their own web sites as in the Scopus database (e.g., *Oncogene*, *Int. J. Cardiol.*), the extreme differences were obtained when analyzing *Bull. Exp. Biol. Med.*, having only 44 documents in press at its web sites, but 571 documents in press in Scopus.

Based on the information available at journals’ web sites, cumulative publication lag caused by delayed print publication of online available in press articles ranged 3.2 (*Bull. Exp. Biol. Med*.) to 781.1 years (*Arab. J. Chem.*) (Fig. 2). More relevant to the decision to submit the manuscript to the respective journal may be the above variable adjusted to the single manuscript. Mean publication lag of a manuscript caused by delayed print publication of online available in press articles ranged from 0.07 years (*Bull. Exp. Biol. Med.*) and 0.34 years (*J. Appl. Polym. Sci.*) to 0.77 years (*Arab. J. Geosci.*) and 1.30 years (*Arab. J. Chem.*). Such significant publication delay corresponds to the one, which can be more easily observed in the Scopus database. The initial search for random in press documents containing the search phrase TITLE-ABS-KEY(a*) revealed 222,985 in press documents. Although less than 18% of them were released before year 2012, some of them were from year 2004 and a single document was claimed to be released even in 1961, suggesting extensive errors of commission. Thus, when analyzing the in press documents revealed by the Scopus searches described in detail below, I randomly checked the credibility of the oldest records. Although many of them were found to be errors (cf. the Tables S1, S2, S3, S4, S5, S6, S7, S8, S9, S10, S11, S12, S13, S14, S15, S16, S17, S18 in Appendix S1), direct search at the journals’ web sites confirmed part of these records spanning up to the year 2006 [21]. Interestingly, even broader timespan was received when analyzing in press records at the web sites of the journals themselves, where two web sites presented in press documents released in 2006, another three web sites contained in press documents released in 2005, and one web site (*Appl. Microbiol. Biotechnol.*) contained even an in press document released online in February 2004 and never published in print. The above phenomenon was absent in *PLoS ONE*, as is in any other online-only journal. Noticeable is also that some in press documents were associated with different year of release in Scopus when compared to the journals’ web sites – e.g., the three papers [22]–[24] released online by *Int. J. Adv. Manuf. Technol.* in 2009, but listed in Scopus as released in 2008, none of them was ever published until the analysis was performed.

### Authors’ Self-citations to Documents In Press

**Figure 4:**
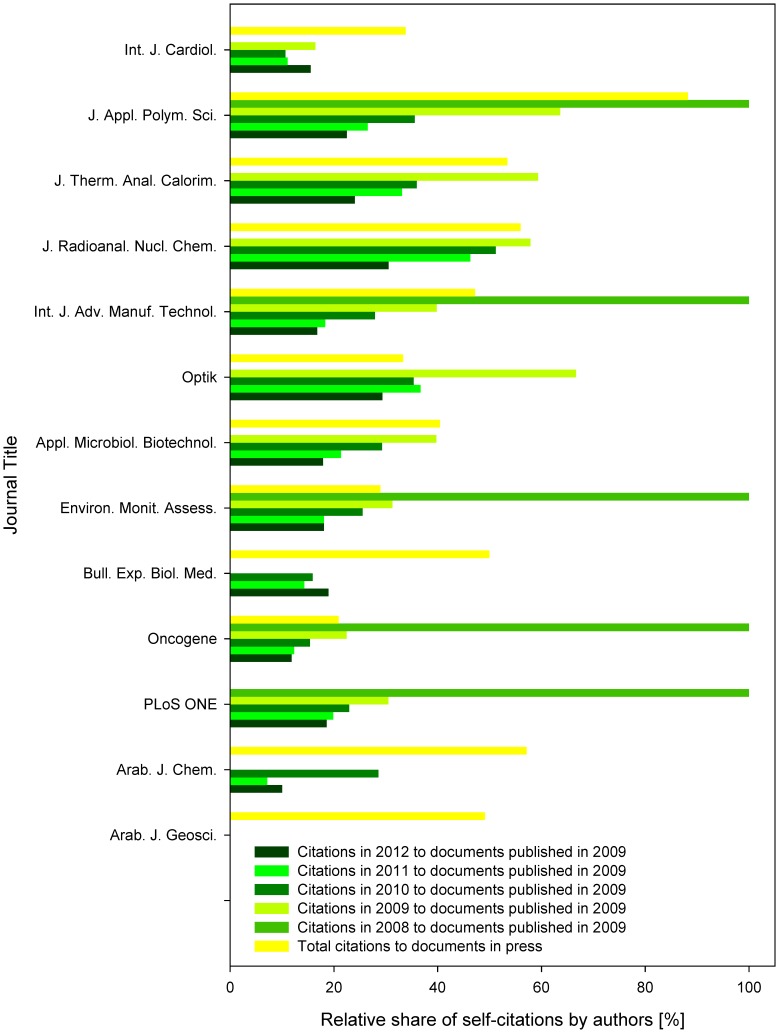
Relative share of authors’ self-citations. The data represent shares of citations, when at least one of the authors of a cited document was the same person as one of the authors of the citing document. Indicated are relative shares of authors’ self-citations among total citations to documents in press, and (annually) among citations in 2008–2012 to documents published in 2009. Except for *Int. J. Cardiol*. cites in 2008 to 2009, all the other zero values indicate absence of the source data in Scopus.

**Figure 5:**
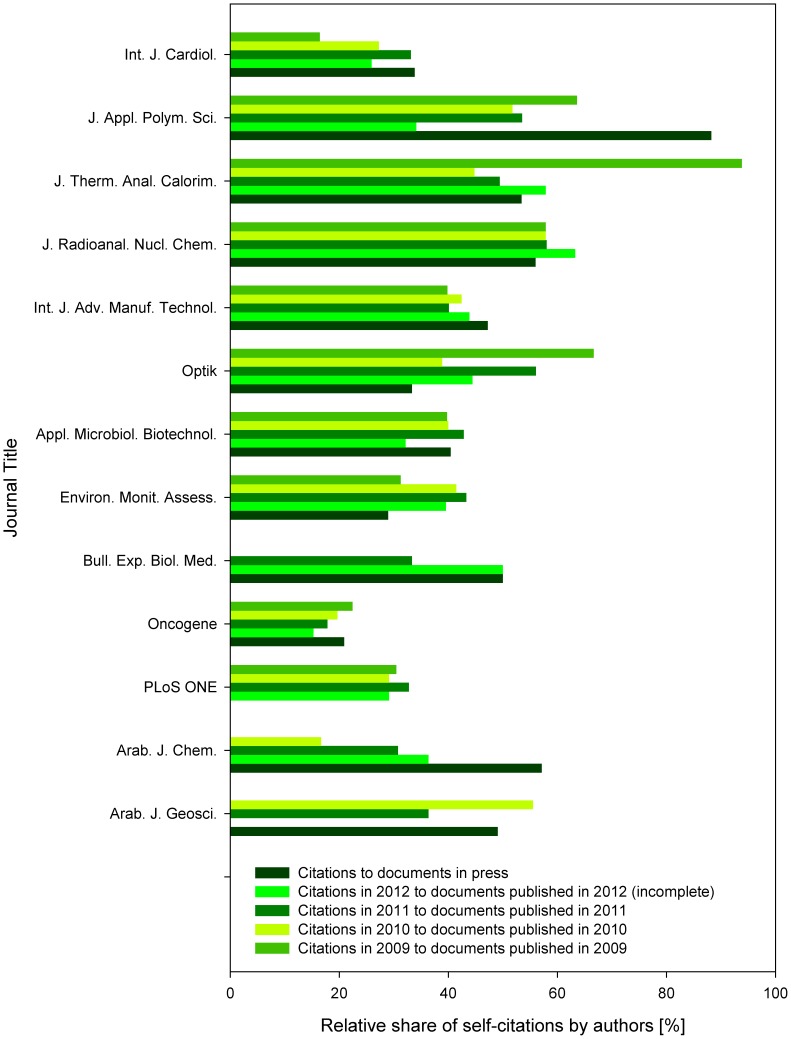
Relative share of authors’ self-citations. The data represent shares of citations, when at least one of the authors of a cited document was the same person as one of the authors of the citing document. Indicated are relative shares of authors’ self-citations among total citations to documents in press, and citations received within the year when the document was assigned to a journal issue (e.g., cites in 2009 to documents published in 2009; analyzed are documents published in 2009–2012). Zero values indicate absence of the source data in Scopus.

When focusing at the level of authors’ self-citations, the highest similarity was found between the level of self-citations to in press articles and citations received within the year when the document was assigned to a journal issue (Fig. 4). The similarity was tested for the 10 journals with available paired data for in press articles (all but *PLoS ONE*) and for cites in 2009 of documents from 2009 (all but *Arab. J. Geosci.* and *Arab. J. Chem.*) (mean_cites in 2009 to docs from 2009_ = 39.7, mean_cites to in press_  = 45.2, paired *t*-test *p*>0.05, t = −0.796, sign test *p*>0.05, r  = 5, n  = 10). For the well-established journals, such as *Oncogene*, the level of authors’ first-year self-citations was found to be very stable over the four-year period. In contrary, journals such as *Arab. J. Chem*. (only recently indexed by Scopus) were subject to strong fluctuations in the number of authors’ self-citations (Fig. 5).

When comparing the annual number of citations received in 2008–2012 to documents published in 2009 with the citations to in press papers, the differences among the tested groups were highly statistically significant (one-way ANOVA *p*<0.01, F  = 12.84, df  = 5). The highest level of authors’ self-citations was received by the self-citations in the year preceding the print edition of the cited articles (e.g., citations in 2008 to articles published in print in 2009). Although this type of citations is rare, when present, in five of the six journals they were based exclusively at the authors’ self-citations. The level of authors’ self-citations in these documents was significantly higher than in those citing the in press documents (Tukey’s *p*<0.01).

Ratio of self-citations to the years following the year of publication of the print document (e.g., citations in 2010 and later to documents published in 2009) were showing a decreasing pattern in most of the examined journals, in the remaining ones the pattern was stagnating (*Int. J. Cardiol*. and *Bull. Exp. Biol. Med*.) (Fig. 4). This corresponded to the increasing significance of differences between the level of authors’ self-citations to these documents when compared to those to articles in press (Tukey’s *p*  = 0.02, 0.04, 0.20 and 0.93 for the level of authors’ self-citations vs. citations in 2012, 2011, 2010 and 2009 to documents published in 2009).

### Journals’ Self-citations to Documents In Press

**Figure 6:**
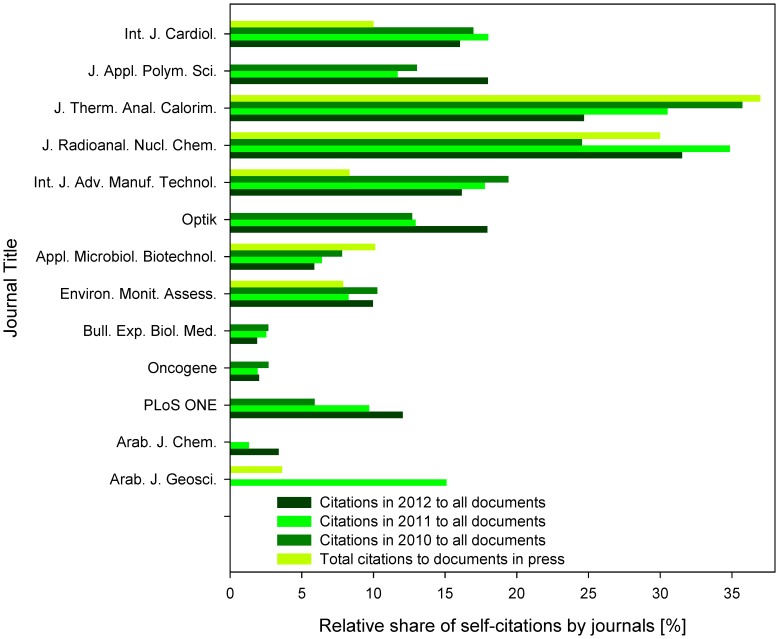
Relative share of journals’ self-citations. The data represent shares of citations, which originate from the same source journal title as the cited document. Indicated is the ratio of journals’ self-citations among the total citations to documents in press and among citations in 2010, 2011 or 2012 to all documents published in the respective journal. Zero values indicate absence of the source data in Scopus.

Two strategies are utilized to determine the order in which the in press documents appear later in the journals’ print issues. Significant part of the journals adopts the first-come-first-serve rule, while many others accelerate the print publication of some papers over the manuscripts accepted earlier. Choice between these two strategies affects strongly the number of journals’ self-citations to their in press documents. Journals sorting in press documents to the print issues based on the first-come-first-serve rule are expected to reach zero journals’ self-citation rate of their documents in press. This was true for *J. Appl. Polym. Sci*., *Optik*, *Bull. Exp. Biol. Med*., *Oncogene* and *Arab. J. Chem*. (Fig. 6). However, the other journals reached up to 37% journals’ self-citation rate (*J. Therm. Anal. Calorim.*), which was even higher than the self-citation rate of documents published in print in the same journal.

The documents published in print received very variable journals’ self-citation rate, reaching 20–40% in *J. Therm. Anal. Calorim.* or *J. Radioanal. Nucl. Chem.*, but less then 5% in *Bull. Exp. Biol. Med*., *Oncogene*, and *Arab. J. Chem*. (Fig. 6). It was possible to observe some trends, such as the doubling of journals’ self-citation rate between 2010 and 2012 in *PLoS ONE* and moderate decreases of the same variable in several other journals (Fig. 6). Detailed analysis of these trends is beyond the scope of this report focusing primarily on the effects of online publication of documents in press.

### Total Citations to Documents in Press

**Figure 7:**
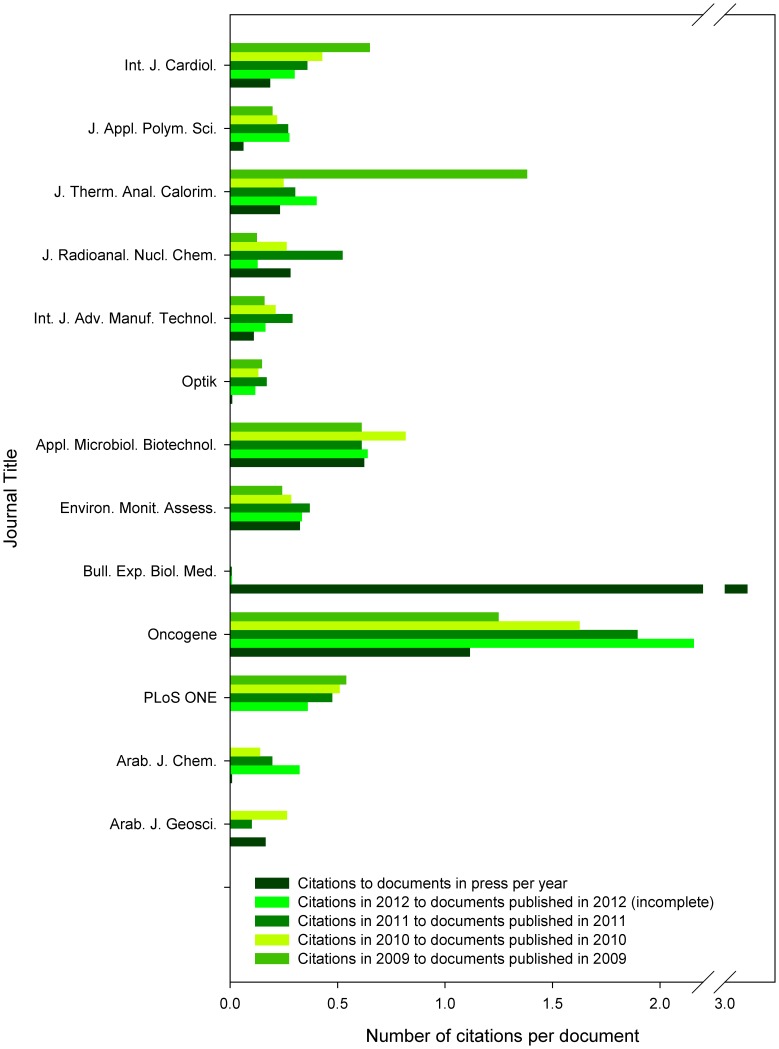
Normalized number of citations received by the documents in press and immediacy indexes of the finalized documents. The data represent citations to documents in press per year, calculated as the number of citations to the documents in press divided by the mean publication lag. For a comparison, immediacy indexes were calculated for the last four years (2009–2012, note that data for 2012 were incomplete at the time of analysis).

Number of citations received by the documents in press per year was very high, similar to those received within the year when the document was assigned to a journal issue, and thus forming substantial part of their immediacy index (calculated as the average number of times an article is cited in the year it is published). Although this index was aimed to indicate publications of the cutting edge research in fields such as medicine or molecular biology, the results show the opposite – even for the highest ranking journal analyzed – *Oncogene* – substantial part of its immediacy index is formed by inclusion of citations to its documents in press (Fig. 7). The extent of the citations to the documents in press is even higher since one has to consider that Scopus does not release citations by documents being still in press, and, naturally, cannot contain citations to documents in press by any documents being still under submission. The difference between the citations received by the documents in press per year and immediacy indexes of the same journals in 2009–2012 were not significant (one-way ANOVA *p*>0.05, F  = 0.05, df  = 4; any Tukey’s pairwise comparisons *p*>0.05).

Interestingly, some journals showed immediacy index changes in time, namely *Int. J. Cardiol.* and *PLoS ONE* experienced gradual decrease of this measure between 2009 and 2012, while *Oncogene* and *Arab. J. Chem*. were experiencing increase in their immediacy indexes (Fig. 7).

## Discussion

Prior to this report, only a single study (published online just two days before this manuscript was submitted) addressed the effects of online first publication of journal articles on scientometric indicators of their source journals. While this study addresses the effects on citations received within the year when the document was assigned to a journal issue and compared them with citations to documents in press, the other report (published coincidentally in PLoS ONE as well [25], addressed the effect of increasing publication lag at the citations utilized for impact factor calculation. Besides these two studies, the only hitherto investigated topics include the publication lag in general [2], [16], [26], the impact of preprint repositories on the citation behavior and visibility of science outputs among the peer scholars [5], [27]–[28], and the effects of the switch from print to dual or online only publication systems [7], [9]–[10]. Also the field of self-citations was extensively covered, although from a different points of view [29]. Journal self-citing and self-cited rates were shown to be inversely correlated with the impact factor [30], and the self-citation rates were shown to be higher during the initial period after publication (as shown also in this report, Fig. 4), and to differ among disciplines [31]–[32].

Here I show that the citation and self-citation patterns to documents in press and to their subsequent print editions share many similar features regardless of the perceived quality of the journals. The analyzed journals include both the well-established ones, such as *Oncogene*, with impact factor according to the Thomson Reuters Journal Citation Reports (JCR) Science Edition 2011 (IF_2011_) 6.4, but also the low-ranking journals such as *Optik* (IF_2011_ = 0.5) or *Bull. Exp. Biol. Med.* (IF_2011_ = 0.3), and the journals only recently indexed in Scopus and Web of Science such as *Arab. J. Chem.* (IF_2011_ = 1.4, JCR-indexed from 2009) and *Arab. J. Geosci.* (IF_2011_ = 1.1, JCR-indexed from 2008). However, some differences were found and are discussed below.

Although the Scopus records provided from major publishers seem to be well updated, some problems occur with data from the smaller publishing houses. *Bull. Exp. Biol. Med.* displayed only 44 documents in press at its web site; however 571 documents in press were indicated by Scopus (Fig. 2). I assume that this happened by the mechanism, which I (to my displeasure) experienced as a guest editor of another journal title, where the journal staff forwarded the abstracts and other data to PubMed just when the papers were in the proofs stage and weeks before they appeared online. One may speculate that the same happened here since *Bull. Exp. Biol. Med.* publishes translations from its Russian-language twin, and thus the publication records are available long before they undergo the translation and further copyediting. With the other journals analyzed, the differences seemed to reflect only the time needed for the implementation of new datasets to the Scopus database, higher frequency of errors was evident only in case of older papers being supposedly still in press.

Another methodical issue was determined with authors’ first-year self-citations. Their levels remained stable in well-established journals, but were found to be fluctuating in the journals only recently indexed by both Scopus and JCR, such as *Arab. J. Chem.* Besides the possibility that the fluctuations reflect the real and strong changes in habits of the authors publishing in the respective journals, there is a possibility that the fluctuations were caused largely by changes in online indexing of accepted manuscripts or by changes of the publisher’s handling with the accepted manuscripts. Changes in publisher’s policies and/or changes in the number of submitted and accepted manuscripts due to the entry of major citation databases may stay behind the observed excessive number of documents in press (when compared to the number of documents published annually). One can speculate that the number of documents in press in these journals is currently substantially higher than couple years ago, and that also the publisher might speeded-up the online post-acceptance release of documents in press, which also contributes strongly to their citedness (otherwise such documents would be cited only by the narrow group of scholars who would know about their existence before their online or print appearance).

All the observed effects caused by citations to documents in press published online project themselves to the immediacy index values. Although the immediacy index is recognized only as a surrogate or complementary measure, its changes may well indicate the changing perception of importance and impact of documents published in the particular journal, especially within the cutting-edge fields of research. This is true especially for the online only journals, which cannot be affected by the phenomenon of citations to documents in press. The single online only journal included in this report, *PLoS ONE*, displayed gradually decreasing immediacy index, which may not necessarily reflect decreasing quality of published papers, but may simply reflect decreasing number of people reading regularly its tables of contents due to the time issues since the number of records published in *PLoS ONE* nearly doubled each year between 2007 and 2012. In the other three journal titles with gradually changing immediacy index (*Int. J. Cardiol*. – decrease, and *Oncogene* and *Arab. J. Chem*. – increase), the immediacy index is strongly affected by citations to the in press articles and the extent of observed immediacy index changes are much smaller than is the share of citations received due to the excessive online presence of documents in press (Fig. 2–3,7). The similarity of the normalized number of citations to documents in press, and citations received within the year when the document was assigned to a journal issue (Fig. 7) reminds me of the conclusions presented by Iain D. Craig et al. [33], who found that preprint availability causes the citation counting process to start earlier although this earlier citation counting did not affect the final magnitude of citations accrued to a journal article.

Concluded, numerous journals across the science disciplines and regardless of their perceived standing within the community publish online hundreds of documents in press, which remain officially unpublished for time periods ranging from couple days to over a year. Cumulative publication lag of a particular journal was found to reach up to enormous 781 years, which strongly affects the citation counts and derived scientometric indicators, predominantly the immediacy index. Once available online, the documents in press are well perceived by the peer scholars and receive citation rates nearly equal to documents published in print. Caution should be taken when performing scientometric evaluation of journals potentially causing long publication lag in a form of long time elapsed between manuscript acceptance and finalization, while supporting the online presence of the in press document at their web sites. Further research should answer the question, on whether the immediacy index should be replaced by an indicator based on the date of first publication (online or in print, whichever comes first) to eliminate the problems analyzed in this report.

## Supporting Information

Appendix S1 **Supporting tables.** Table S1. Number of documents per year. The Scopus database was searched for any documents published in the indicated journals in the respective years. Documents in press are included in these data, assigned are to the year of their on-line publication. Data are not corrected for any database errors. Arab. J. Geosci. is included in the Scopus database only since 2009. Table S2. Number of particular document types published by each respective journal according to Scopus. Counted are all documents included in Scopus until the analysis date (cf. Table S1). Table S3. Publication years of in press documents included in the Scopus database at the day of analysis (cf. Table S1). Data are not corrected for any database errors, however the outliers’ credibility was manually checked at the journals’ web sites, results of these manual checks are shown in brackets, when the first number indicates number of verified in press documents, while the second number indicates the number of documents erroneously shown as documents in press in the Scopus database, but being already published). Besides that, among the eight verified in press documents published by Int. J. Adv. Manuf. Technol. are three, for which the year of on-line publication does not correspond to the year indicated in Scopus. Table S4. Number of self-citations by the respective journals. Table S5. Publication years of documents citing the documents in press present in the Scopus database at the day of analysis (cf. Table S1). Table S6. Publication years of documents citing the documents published in the 2013 volumes of the respective journals and present in the Scopus database at the day of analysis (cf. Table S1). Table S7. Publication years of documents citing the documents published in the 2012 volumes of the respective journals and present in the Scopus database at the day of analysis (cf. Table S1). Table S8. Publication years of documents citing the documents published in the 2011 volumes of the respective journals and present in the Scopus database at the day of analysis (cf. Table S1). Table S9. Publication years of documents citing the documents published in the 2010 volumes of the respective journals and present in the Scopus database at the day of analysis (cf. Table S1). Table S10. Publication years of documents citing the documents published in the 2009 volumes of the respective journals and present in the Scopus database at the day of analysis (cf. Table S1). Table S11. Publication years of documents citing the documents in press present in the Scopus database at the day of analysis (cf. Table S1). Self-citations by any of the authors are excluded. Table S12. Publication years of documents citing the documents published in the 2013 volumes of the respective journals and present in the Scopus database at the day of analysis (cf. Table S1). Self-citations by any of the authors are excluded. Table S13. Publication years of documents citing the documents published in the 2012 volumes of the respective journals and present in the Scopus database at the day of analysis (cf. Table S1). Self-citations by any of the authors are excluded. Table S14. Publication years of documents citing the documents published in the 2011 volumes of the respective journals and present in the Scopus database at the day of analysis (cf. Table S1). Self-citations by any of the authors are excluded. Table S15. Publication years of documents citing the documents published in the 2010 volumes of the respective journals and present in the Scopus database at the day of analysis (cf. Table S1). Self-citations by any of the authors are excluded. Table S16. Publication years of documents citing the documents published in the 2009 volumes of the respective journals and present in the Scopus database at the day of analysis (cf. Table S1). Self-citations by any of the authors are excluded. Table S17. Total number of citations (first three columns) and number of citations excluding self-citations by the journal (last three columns) citing any documents published in the indicated journals and included in the Scopus database at the day of analysis (cf. Table S1). The Scopus database was searched for any documents published in the indicated journals in the respective years. Citations to documents in press are included in these data as well. Table S18. Source web sites, dates of analyses, number of in press documents according to journals’ web sites, and dates of on-line publication of newest and oldest documents remaining in press at the analysis date. For journals published by Springer Verlag, the old version of SpringerLink was used since it allowed easier evaluation of the age of published in press documents when compared to its updated version. As a disadvantage, the old version of SpringerLink was not updated during the month preceding the analyses (as indicated in the Analysis date column). Data from these analyses were used for calculation of mean age of in press documents in each of the respective journals.
(DOCX)
