# Supplementary material for: Effects of Print Publication Lag in Dual Format Journals on Scientometric Indicators
Source: PLoS One. 2013 Apr 3;8(4):e59877. doi: 10.1371/journal.pone.0059877 (PMC3616011; doi:10.1371/journal.pone.0059877)
Supplement: Appendix S1 — Supporting tables. Table S1. Number of documents per year. The Scopus database was searched for any documents published in the indicated journals in the respective years. Documents in press are included in these data, assigned are to the year of their on-line publication. Data are not corrected for any database errors. Arab. J. Geosci. is included in the Scopus database only since 2009. Table S2. Number of particular document types published by each respective journal according to Scopus. Counted are all documents included in Scopus until the analysis date (cf. Table S1). Table S3. Publication years of in press documents included in the Scopus database at the day of analysis (cf. Table S1). Data are not corrected for any database errors, however the outliers’ credibility was manually checked at the journals’ web sites, results of these manual checks are shown in brackets, when the first number indicates number of verified in press documents, while the second number indicates the number of documents erroneously shown as documents in press in the Scopus database, but being already published). Besides that, among the eight verified in press documents published by Int. J. Adv. Manuf. Technol. are three, for which the year of on-line publication does not correspond to the year indicated in Scopus. Table S4. Number of self-citations by the respective journals. Table S5. Publication years of documents citing the documents in press present in the Scopus database at the day of analysis (cf. Table S1). Table S6. Publication years of documents citing the documents published in the 2013 volumes of the respective journals and present in the Scopus database at the day of analysis (cf. Table S1). Table S7. Publication years of documents citing the documents published in the 2012 volumes of the respective journals and present in the Scopus database at the day of analysis (cf. Table S1). Table S8. Publication years of documents citing the documents published in the 2011 volumes of t [file pone.0059877.s001.docx]

## SUPPORTING INFORMATION

(source data)

## Title: Effects of print publication lag in dual format journals on scientometric indicators

## Author and Affiliation: Petr Heneberg^1,*^

^1^Third Faculty of Medicine, Charles University in Prague, Prague, Czech Republic

* E-mail: petr.heneberg@lf3.cuni.cz

**Table S1.** Number of documents per year. The Scopus database was searched for any documents published in the indicated journals in the respective years. Documents in press are included in these data, assigned are to the year of their on-line publication. Data are not corrected for any database errors. Arab. J. Geosci. is included in the Scopus database only since 2009.

| Journal Title | Analysis date | 2013 | 2012 | 2011 | 2010 | 2009 | 2008 | 2007 | 2006 | 2005 | 2004 | 2003 |
| --- | --- | --- | --- | --- | --- | --- | --- | --- | --- | --- | --- | --- |
| Int. J. Cardiol. | 20.12.2012 | 28 | 940 | 1161 | 1027 | 851 | 1037 | 1108 | 820 | 682 | 432 | 309 |
| J. Appl. Polym. Sci. | 20.12.2012 | 506 | 2907 | 1774 | 1802 | 1821 | 2052 | 2083 | 2460 | 1142 | 1702 | 1656 |
| J. Therm. Anal. Calorim. | 20.12.2012 | 0 | 948 | 802 | 631 | 595 | 583 | 573 | 481 | 468 | 383 | 431 |
| J. Radioanal. Nucl. Chem. | 20.12.2012 | 0 | 927 | 625 | 444 | 519 | 474 | 469 | 397 | 444 | 418 | 440 |
| Int. J. Adv. Manuf. Technol. | 20.12.2012 | 0 | 1225 | 666 | 659 | 713 | 547 | 525 | 638 | 389 | 225 | 234 |
| Optik | 21.12.2012 | 21 | 1073 | 495 | 410 | 182 | 144 | 104 | 100 | 96 | 99 | 116 |
| Appl. Microbiol. Biotechnol. | 21.12.2012 | 0 | 1102 | 710 | 704 | 432 | 502 | 654 | 541 | 326 | 317 | 308 |
| Environ. Monit. Assess. | 21.12.2012 | 0 | 1044 | 627 | 674 | 569 | 460 | 476 | 386 | 239 | 185 | 187 |
| Bull. Exp. Biol. Med. | 21.12.2012 | 0 | 577 | 598 | 263 | 433 | 427 | 411 | 394 | 404 | 352 | 400 |
| Oncogene | 21.12.2012 | 0 | 1010 | 476 | 616 | 429 | 775 | 816 | 800 | 866 | 1035 | 1021 |
| PLoS ONE | 20.12.2012 | 0 | 21145 | 13945 | 7692 | 4561 | 2747 | 1366 | 137 | 0 | 0 | 0 |
| Arab. J. Chem. | 22.12.2012 | 0 | 306 | 321 | 90 | 17 | 0 | 0 | 0 | 0 | 0 | 0 |
| Arab. J. Geosci. | 22.12.2012 | 0 | 170 | 333 | 104 | 5 | 0 | 0 | 0 | 0 | 0 | 0 |

**Table S2.** Number of particular document types published by each respective journal according to Scopus. Counted are all documents included in Scopus until the analysis date (cf. Table S1).

| Journal Title | Article in Press | Article | Conference Paper | Review | Letter | Note | Editorial | Short Survey | Erratum | Conference Review | Undefined |
| --- | --- | --- | --- | --- | --- | --- | --- | --- | --- | --- | --- |
| Int. J. Cardiol. | 1196 | 9536 | 229 | 667 | 2272 | 49 | 367 | 65 | 60 |  | 80 |
| J. Appl. Polym. Sci. | 813 | 33561 | 340 | 70 |  | 1 | 17 | 1 | 73 | 8 | 2966 |
| J. Therm. Anal. Calorim. | 699 | 4708 | 2438 | 40 | 7 | 2 | 65 |  | 8 | 7 |  |
| J. Radioanal. Nucl. Chem. | 735 | 10005 | 2696 | 137 | 15 | 21 | 69 | 18 | 106 |  | 453 |
| Int. J. Adv. Manuf. Technol. | 689 | 6225 | 38 | 72 |  |  | 21 |  | 33 |  |  |
| Optik | 626 | 2156 |  | 8 |  | 3 |  | 1 | 2 |  | 78 |
| Appl. Microbiol. Biotechnol. | 608 | 8235 |  | 241 |  |  | 12 | 797 | 104 |  | 653 |
| Environ. Monit. Assess. | 591 | 4998 | 703 | 132 |  |  |  |  | 47 |  |  |
| Bull. Exp. Biol. Med. | 571 | 21653 |  | 144 |  |  | 2 | 1 | 18 |  | 3194 |
| Oncogene | 573 | 14331 | 11 | 1262 | 10 | 16 | 71 | 26 | 205 |  | 28 |
| PLoS ONE |  | 51215 |  | 371 |  |  | 1 |  | 6 |  |  |
| Arab. J. Chem. | 540 | 182 |  | 11 |  |  |  |  | 1 |  |  |
| Arab. J. Geosci. | 465 | 143 |  | 2 |  |  | 1 |  | 1 |  |  |

**Table S3.** Publication years of in press documents included in the Scopus database at the day of analysis (cf. Table S1). Data are not corrected for any database errors, however the outliers’ credibility was manually checked at the journals’ web sites, results of these manual checks are shown in brackets, when the first number indicates number of verified in press documents, while the second number indicates the number of documents erroneously shown as documents in press in the Scopus database, but being already published). Besides that, among the eight verified in press documents published by Int. J. Adv. Manuf. Technol. are three, for which the year of on-line publication does not correspond to the year indicated in Scopus.

| Journal Title | 2013 | 2012 | 2011 | 2010 | 2009 | 2008 | 2007 | 2006 | 2005 | 2004 | 1961 |
| --- | --- | --- | --- | --- | --- | --- | --- | --- | --- | --- | --- |
| Int. J. Cardiol. |  | 947 | 248 | 1 |  |  |  |  |  |  |  |
| J. Appl. Polym. Sci. |  | 794 | 19 |  |  |  |  |  |  |  |  |
| J. Therm. Anal. Calorim. |  | 459 | 221 | 11 | 6 | 1 (1,0) | 1 (0,1) |  |  |  |  |
| J. Radioanal. Nucl. Chem. |  | 545 | 115 | 11 | 61 | 3 (2,1) |  |  |  |  |  |
| Int. J. Adv. Manuf. Technol. |  | 629 | 39 | 3 | 7 | 11 (8,3) |  |  |  |  |  |
| Optik |  | 613 | 13 |  |  |  |  |  |  |  |  |
| Appl. Microbiol. Biotechnol. |  | 529 | 62 |  | 1 | 15 |  | 1 (1,0) |  |  |  |
| Environ. Monit. Assess. |  | 500 | 22 | 14 | 41 | 10 | 4 (3,1) |  |  |  |  |
| Bull. Exp. Biol. Med. |  | 355 | 215 | 1 |  |  |  |  |  |  |  |
| Oncogene |  | 549 | 14 | 1 | 9 (0,9) |  |  |  |  |  |  |
| PLoS ONE |  |  |  |  |  |  |  |  |  |  |  |
| Arab. J. Chem. |  | 238 | 255 | 47 |  |  |  |  |  |  |  |
| Arab. J. Geosci. |  | 166 | 224 | 70 | 5 |  |  |  |  |  |  |
| Random sample ("a*") | 6379 | 176968 | 23574 | 7525 | 4089 | 3967 | 428 | 44 | 5 | 5 | 1 |

**Table S4.** Number of self-citations by the respective journals.

| Journal Title | Number of self-citations by the journal | Relative number of self-citations by the journal [%] |
| --- | --- | --- |
| Int. J. Cardiol. | 13 | 10.00 |
| J. Appl. Polym. Sci. | 0 | 0.00 |
| J. Therm. Anal. Calorim. | 27 | 36.99 |
| J. Radioanal. Nucl. Chem. | 15 | 30.00 |
| Int. J. Adv. Manuf. Technol. | 3 | 8.33 |
| Optik | 0 | 0.00 |
| Appl. Microbiol. Biotechnol. | 9 | 10.11 |
| Environ. Monit. Assess. | 6 | 7.89 |
| Bull. Exp. Biol. Med. | 0 | 0.00 |
| Oncogene | 0 | 0.00 |
| PLoS ONE | N/A | N/A |
| Arab. J. Chem. | 0 | 0.00 |
| Arab. J. Geosci. | 2 | 3.64 |

**Table S5.** Publication years of documents citing the documents in press present in the Scopus database at the day of analysis (cf. Table S1).

| Journal Title | Documents | Total cites | 2013 | 2012 | 2011 | 2010 | 2009 | 2008 |
| --- | --- | --- | --- | --- | --- | --- | --- | --- |
| Int. J. Cardiol. | 1196 | 130 | 4 | 126 | 0 | 0 | 0 | 0 |
| J. Appl. Polym. Sci. | 813 | 17 | 4 | 13 | 0 | 0 | 0 | 0 |
| J. Therm. Anal. Calorim. | 699 | 73 | 4 | 56 | 9 | 3 | 1 | 0 |
| J. Radioanal. Nucl. Chem. | 735 | 50 | 0 | 29 | 12 | 9 | 0 | 0 |
| Int. J. Adv. Manuf. Technol. | 689 | 36 | 5 | 17 | 7 | 3 | 4 | 0 |
| Optik | 626 | 3 | 1 | 2 | 0 | 0 | 0 | 0 |
| Appl. Microbiol. Biotechnol. | 608 | 89 | 6 | 68 | 7 | 6 | 2 | 0 |
| Environ. Monit. Assess. | 591 | 76 | 1 | 41 | 21 | 6 | 6 | 1 |
| Bull. Exp. Biol. Med. | 571 | 10 | 2 | 8 | 0 | 0 | 0 | 0 |
| Oncogene | 573 | 268 | 5 | 245 | 14 | 1 | 3 | 0 |
| PLoS ONE | 0 | 0 | 0 | 0 | 0 | 0 | 0 | 0 |
| Arab. J. Chem. | 540 | 7 | 3 | 3 | 1 | 0 | 0 | 0 |
| Arab. J. Geosci. | 465 | 55 | 0 | 37 | 17 | 1 | 0 | 0 |

**Table S6.** Publication years of documents citing the documents published in the 2013 volumes of the respective journals and present in the Scopus database at the day of analysis (cf. Table S1).

| Journal Title | Documents | Total cites | 2013 | 2012 | 2011 | 2010 | 2009 | 2008 |
| --- | --- | --- | --- | --- | --- | --- | --- | --- |
| Int. J. Cardiol. | 28 | 0 | 0 | 0 | 0 | 0 | 0 | 0 |
| J. Appl. Polym. Sci. | 506 | 7 | 0 | 7 | 0 | 0 | 0 | 0 |
| J. Therm. Anal. Calorim. | 0 | 0 | 0 | 0 | 0 | 0 | 0 | 0 |
| J. Radioanal. Nucl. Chem. | 0 | 0 | 0 | 0 | 0 | 0 | 0 | 0 |
| Int. J. Adv. Manuf. Technol. | 0 | 0 | 0 | 0 | 0 | 0 | 0 | 0 |
| Optik | 21 | 0 | 0 | 0 | 0 | 0 | 0 | 0 |
| Appl. Microbiol. Biotechnol. | 0 | 0 | 0 | 0 | 0 | 0 | 0 | 0 |
| Environ. Monit. Assess. | 0 | 0 | 0 | 0 | 0 | 0 | 0 | 0 |
| Bull. Exp. Biol. Med. | 0 | 0 | 0 | 0 | 0 | 0 | 0 | 0 |
| Oncogene | 0 | 0 | 0 | 0 | 0 | 0 | 0 | 0 |
| PLoS ONE | 0 | 0 | 0 | 0 | 0 | 0 | 0 | 0 |
| Arab. J. Chem. | 0 | 0 | 0 | 0 | 0 | 0 | 0 | 0 |
| Arab. J. Geosci. | 0 | 0 | 0 | 0 | 0 | 0 | 0 | 0 |

**Table S7.** Publication years of documents citing the documents published in the 2012 volumes of the respective journals and present in the Scopus database at the day of analysis (cf. Table S1).

| Journal Title | Documents | Total cites | 2013 | 2012 | 2011 | 2010 | 2009 | 2008 |
| --- | --- | --- | --- | --- | --- | --- | --- | --- |
| Int. J. Cardiol. | 1887 | 605 | 17 | 567 | 21 | 0 | 0 | 0 |
| J. Appl. Polym. Sci. | 2113 | 682 | 94 | 583 | 5 | 0 | 0 | 0 |
| J. Therm. Anal. Calorim. | 489 | 209 | 11 | 197 | 1 | 0 | 0 | 0 |
| J. Radioanal. Nucl. Chem. | 382 | 53 | 4 | 49 | 0 | 0 | 0 | 0 |
| Int. J. Adv. Manuf. Technol. | 596 | 120 | 17 | 98 | 5 | 0 | 0 | 0 |
| Optik | 460 | 65 | 6 | 54 | 5 | 0 | 0 | 0 |
| Appl. Microbiol. Biotechnol. | 573 | 389 | 19 | 367 | 3 | 0 | 0 | 0 |
| Environ. Monit. Assess. | 544 | 204 | 10 | 182 | 11 | 1 | 0 | 0 |
| Bull. Exp. Biol. Med. | 222 | 2 | 0 | 2 | 0 | 0 | 0 | 0 |
| Oncogene | 461 | 1047 | 33 | 995 | 19 | 0 | 0 | 0 |
| PLoS ONE | 21145 | 7920 | 271 | 7647 | 2 | 0 | 0 | 0 |
| Arab. J. Chem. | 68 | 23 | 0 | 22 | 1 | 0 | 0 | 0 |
| Arab. J. Geosci. | 4 | 0 | 0 | 0 | 0 | 0 | 0 | 0 |

**Table S8.** Publication years of documents citing the documents published in the 2011 volumes of the respective journals and present in the Scopus database at the day of analysis (cf. Table S1).

| Journal Title | Documents | Total cites | 2013 | 2012 | 2011 | 2010 | 2009 | 2008 |
| --- | --- | --- | --- | --- | --- | --- | --- | --- |
| Int. J. Cardiol. | 1409 | 1902 | 7 | 1373 | 507 | 15 | 0 | 0 |
| J. Appl. Polym. Sci. | 1774 | 2769 | 222 | 2064 | 480 | 3 | 0 | 0 |
| J. Therm. Anal. Calorim. | 581 | 897 | 24 | 697 | 176 | 0 | 0 | 0 |
| J. Radioanal. Nucl. Chem. | 510 | 856 | 12 | 577 | 267 | 0 | 0 | 0 |
| Int. J. Adv. Manuf. Technol. | 627 | 956 | 57 | 710 | 182 | 3 | 1 | 1 |
| Optik | 482 | 342 | 23 | 236 | 82 | 1 | 0 | 0 |
| Appl. Microbiol. Biotechnol. | 648 | 2280 | 57 | 1824 | 397 | 2 | 0 | 0 |
| Environ. Monit. Assess. | 605 | 974 | 21 | 720 | 224 | 9 | 0 | 0 |
| Bull. Exp. Biol. Med. | 383 | 41 | 4 | 34 | 3 | 0 | 0 | 0 |
| Oncogene | 462 | 3597 | 36 | 2681 | 876 | 4 | 0 | 0 |
| PLoS ONE | 13945 | 47319 | 724 | 39952 | 6636 | 7 | 0 | 0 |
| Arab. J. Chem. | 66 | 85 | 0 | 72 | 13 | 0 | 0 | 0 |
| Arab. J. Geosci. | 109 | 51 | 5 | 33 | 11 | 2 | 0 | 0 |

**Table S9.** Publication years of documents citing the documents published in the 2010 volumes of the respective journals and present in the Scopus database at the day of analysis (cf. Table S1).

| Journal Title | Documents | Total cites | 2013 | 2012 | 2011 | 2010 | 2009 | 2008 |
| --- | --- | --- | --- | --- | --- | --- | --- | --- |
| Int. J. Cardiol. | 1027 | 4149 | 16 | 1891 | 1795 | 440 | 7 | 0 |
| J. Appl. Polym. Sci. | 1802 | 5382 | 185 | 2713 | 2089 | 394 | 1 | 0 |
| J. Therm. Anal. Calorim. | 620 | 2246 | 32 | 1171 | 889 | 154 | 0 | 0 |
| J. Radioanal. Nucl. Chem. | 433 | 1454 | 14 | 552 | 774 | 114 | 0 | 0 |
| Int. J. Adv. Manuf. Technol. | 656 | 2136 | 46 | 1106 | 845 | 139 | 0 | 0 |
| Optik | 410 | 549 | 15 | 248 | 232 | 54 | 0 | 0 |
| Appl. Microbiol. Biotechnol. | 704 | 5946 | 82 | 2912 | 2377 | 575 | 0 | 0 |
| Environ. Monit. Assess. | 660 | 2026 | 28 | 1021 | 787 | 188 | 2 | 0 |
| Bull. Exp. Biol. Med. | 262 | 124 | 3 | 78 | 43 | 0 | 0 | 0 |
| Oncogene | 616 | 9385 | 74 | 4399 | 3910 | 1002 | 0 | 0 |
| PLoS ONE | 7692 | 60450 | 385 | 31738 | 24401 | 3924 | 2 | 0 |
| Arab. J. Chem. | 43 | 133 | 2 | 78 | 47 | 6 | 0 | 0 |
| Arab. J. Geosci. | 34 | 70 | 2 | 34 | 25 | 9 | 0 | 0 |

**Table S10.** Publication years of documents citing the documents published in the 2009 volumes of the respective journals and present in the Scopus database at the day of analysis (cf. Table S1).

| Journal Title | Documents | Total cites | 2013 | 2012 | 2011 | 2010 | 2009 | 2008 |
| --- | --- | --- | --- | --- | --- | --- | --- | --- |
| Int. J. Cardiol. | 852 | 6502 | 13 | 1403 | 2212 | 2319 | 554 | 1 |
| J. Appl. Polym. Sci. | 1821 | 8511 | 222 | 3037 | 2896 | 1995 | 360 | 1 |
| J. Therm. Anal. Calorim. | 89 | 3005 | 35 | 979 | 1048 | 820 | 123 | 0 |
| J. Radioanal. Nucl. Chem. | 458 | 1473 | 24 | 383 | 585 | 424 | 57 | 0 |
| Int. J. Adv. Manuf. Technol. | 706 | 3410 | 56 | 1258 | 1197 | 785 | 113 | 1 |
| Optik | 182 | 351 | 5 | 109 | 128 | 82 | 27 | 0 |
| Appl. Microbiol. Biotechnol. | 431 | 4948 | 43 | 1667 | 1690 | 1284 | 264 | 0 |
| Environ. Monit. Assess. | 528 | 2806 | 32 | 1018 | 957 | 670 | 128 | 1 |
| Bull. Exp. Biol. Med. | 433 | 220 | 3 | 95 | 77 | 44 | 1 | 0 |
| Oncogene | 428 | 8882 | 63 | 2789 | 2974 | 2520 | 535 | 1 |
| PLoS ONE | 4561 | 64474 | 271 | 21757 | 23564 | 16417 | 2464 | 1 |
| Arab. J. Chem. | 17 | 34 | 3 | 10 | 14 | 7 | 0 | 0 |
| Arab. J. Geosci. | N/A | N/A | N/A | N/A | N/A | N/A | N/A | N/A |

**Table S11.** Publication years of documents citing the documents in press present in the Scopus database at the day of analysis (cf. Table S1). Self-citations by any of the authors are excluded.

| Journal Title | Documents | Total cites | 2013 | 2012 | 2011 | 2010 | 2009 | 2008 |
| --- | --- | --- | --- | --- | --- | --- | --- | --- |
| Int. J. Cardiol. | 1196 | 86 | 2 | 84 | 0 | 0 | 0 | 0 |
| J. Appl. Polym. Sci. | 813 | 2 | 0 | 2 | 0 | 0 | 0 | 0 |
| J. Therm. Anal. Calorim. | 699 | 34 | 3 | 22 | 5 | 3 | 1 | 0 |
| J. Radioanal. Nucl. Chem. | 735 | 22 | 0 | 10 | 9 | 3 | 0 | 0 |
| Int. J. Adv. Manuf. Technol. | 689 | 19 | 1 | 8 | 5 | 2 | 3 | 0 |
| Optik | 626 | 2 | 1 | 1 | 0 | 0 | 0 | 0 |
| Appl. Microbiol. Biotechnol. | 608 | 53 | 3 | 35 | 7 | 6 | 2 | 0 |
| Environ. Monit. Assess. | 591 | 54 | 1 | 25 | 19 | 6 | 3 | 0 |
| Bull. Exp. Biol. Med. | 571 | 5 | 0 | 5 | 0 | 0 | 0 | 0 |
| Oncogene | 573 | 212 | 5 | 193 | 10 | 1 | 3 | 0 |
| PLoS ONE | 0 | 0 | 0 | 0 | 0 | 0 | 0 | 0 |
| Arab. J. Chem. | 540 | 3 | 2 | 1 | 0 | 0 | 0 | 0 |
| Arab. J. Geosci. | 465 | 28 | 0 | 21 | 7 | 0 | 0 | 0 |

**Table S12.** Publication years of documents citing the documents published in the 2013 volumes of the respective journals and present in the Scopus database at the day of analysis (cf. Table S1). Self-citations by any of the authors are excluded.

| Journal Title | Documents | Total cites | 2013 | 2012 | 2011 | 2010 | 2009 | 2008 |
| --- | --- | --- | --- | --- | --- | --- | --- | --- |
| Int. J. Cardiol. | 28 | 0 | 0 | 0 | 0 | 0 | 0 | 0 |
| J. Appl. Polym. Sci. | 506 | 2 | 0 | 2 | 0 | 0 | 0 | 0 |
| J. Therm. Anal. Calorim. | 0 | 0 | 0 | 0 | 0 | 0 | 0 | 0 |
| J. Radioanal. Nucl. Chem. | 0 | 0 | 0 | 0 | 0 | 0 | 0 | 0 |
| Int. J. Adv. Manuf. Technol. | 0 | 0 | 0 | 0 | 0 | 0 | 0 | 0 |
| Optik | 21 | 0 | 0 | 0 | 0 | 0 | 0 | 0 |
| Appl. Microbiol. Biotechnol. | 0 | 0 | 0 | 0 | 0 | 0 | 0 | 0 |
| Environ. Monit. Assess. | 0 | 0 | 0 | 0 | 0 | 0 | 0 | 0 |
| Bull. Exp. Biol. Med. | 0 | 0 | 0 | 0 | 0 | 0 | 0 | 0 |
| Oncogene | 0 | 0 | 0 | 0 | 0 | 0 | 0 | 0 |
| PLoS ONE | 0 | 0 | 0 | 0 | 0 | 0 | 0 | 0 |
| Arab. J. Chem. | 0 | 0 | 0 | 0 | 0 | 0 | 0 | 0 |
| Arab. J. Geosci. | 0 | 0 | 0 | 0 | 0 | 0 | 0 | 0 |

**Table S13.** Publication years of documents citing the documents published in the 2012 volumes of the respective journals and present in the Scopus database at the day of analysis (cf. Table S1). Self-citations by any of the authors are excluded.

| Journal Title | Documents | Total cites | 2013 | 2012 | 2011 | 2010 | 2009 | 2008 |
| --- | --- | --- | --- | --- | --- | --- | --- | --- |
| Int. J. Cardiol. | 1887 | 420 | 11 | 400 | 9 | 0 | 0 | 0 |
| J. Appl. Polym. Sci. | 2113 | 384 | 61 | 322 | 1 | 0 | 0 | 0 |
| J. Therm. Anal. Calorim. | 489 | 83 | 6 | 76 | 1 | 0 | 0 | 0 |
| J. Radioanal. Nucl. Chem. | 382 | 18 | 2 | 16 | 0 | 0 | 0 | 0 |
| Int. J. Adv. Manuf. Technol. | 596 | 55 | 8 | 43 | 4 | 0 | 0 | 0 |
| Optik | 460 | 30 | 2 | 28 | 0 | 0 | 0 | 0 |
| Appl. Microbiol. Biotechnol. | 573 | 249 | 12 | 236 | 1 | 0 | 0 | 0 |
| Environ. Monit. Assess. | 544 | 110 | 6 | 98 | 6 | 0 | 0 | 0 |
| Bull. Exp. Biol. Med. | 222 | 1 | 0 | 1 | 0 | 0 | 0 | 0 |
| Oncogene | 461 | 843 | 27 | 806 | 10 | 0 | 0 | 0 |
| PLoS ONE | 21324 | 5465 | 204 | 5259 | 2 | 0 | 0 | 0 |
| Arab. J. Chem. | 68 | 14 | 0 | 13 | 1 | 0 | 0 | 0 |
| Arab. J. Geosci. | 4 | 0 | 0 | 0 | 0 | 0 | 0 | 0 |

**Table S14.** Publication years of documents citing the documents published in the 2011 volumes of the respective journals and present in the Scopus database at the day of analysis (cf. Table S1). Self-citations by any of the authors are excluded.

| Journal Title | Documents | Total cites | 2013 | 2012 | 2011 | 2010 | 2009 | 2008 |
| --- | --- | --- | --- | --- | --- | --- | --- | --- |
| Int. J. Cardiol. | 1409 | 1396 | 4 | 1040 | 339 | 13 | 0 | 0 |
| J. Appl. Polym. Sci. | 1774 | 1737 | 164 | 1349 | 223 | 1 | 0 | 0 |
| J. Therm. Anal. Calorim. | 581 | 578 | 13 | 476 | 89 | 0 | 0 | 0 |
| J. Radioanal. Nucl. Chem. | 510 | 490 | 6 | 372 | 112 | 0 | 0 | 0 |
| Int. J. Adv. Manuf. Technol. | 627 | 685 | 47 | 523 | 109 | 2 | 1 | 1 |
| Optik | 482 | 182 | 7 | 139 | 36 | 0 | 0 | 0 |
| Appl. Microbiol. Biotechnol. | 648 | 1591 | 44 | 1320 | 227 | 0 | 0 | 0 |
| Environ. Monit. Assess. | 605 | 694 | 19 | 545 | 127 | 3 | 0 | 0 |
| Bull. Exp. Biol. Med. | 383 | 24 | 3 | 19 | 2 | 0 | 0 | 0 |
| Oncogene | 462 | 3080 | 33 | 2325 | 720 | 2 | 0 | 0 |
| PLoS ONE | 13945 | 35308 | 572 | 30267 | 4463 | 6 | 0 | 0 |
| Arab. J. Chem. | 66 | 67 | 0 | 58 | 9 | 0 | 0 | 0 |
| Arab. J. Geosci. | 109 | 35 | 5 | 23 | 7 | 0 | 0 | 0 |

**Table S15.** Publication years of documents citing the documents published in the 2010 volumes of the respective journals and present in the Scopus database at the day of analysis (cf. Table S1). Self-citations by any of the authors are excluded.

| Journal Title | Documents | Total cites | 2013 | 2012 | 2011 | 2010 | 2009 | 2008 |
| --- | --- | --- | --- | --- | --- | --- | --- | --- |
| Int. J. Cardiol. | 1027 | 3446 | 13 | 1616 | 1493 | 320 | 3 | 1 |
| J. Appl. Polym. Sci. | 1802 | 3679 | 137 | 1989 | 1363 | 190 | 0 | 0 |
| J. Therm. Anal. Calorim. | 620 | 1547 | 15 | 853 | 594 | 85 | 0 | 0 |
| J. Radioanal. Nucl. Chem. | 433 | 774 | 10 | 335 | 381 | 48 | 0 | 0 |
| Int. J. Adv. Manuf. Technol. | 656 | 1565 | 38 | 850 | 597 | 80 | 0 | 0 |
| Optik | 410 | 368 | 7 | 182 | 146 | 33 | 0 | 0 |
| Appl. Microbiol. Biotechnol. | 704 | 4494 | 66 | 2313 | 1770 | 345 | 0 | 0 |
| Environ. Monit. Assess. | 660 | 1609 | 21 | 863 | 615 | 110 | 0 | 0 |
| Bull. Exp. Biol. Med. | 262 | 97 | 3 | 62 | 32 | 0 | 0 | 0 |
| Oncogene | 616 | 8073 | 67 | 3826 | 3375 | 805 | 0 | 0 |
| PLoS ONE | 7692 | 47468 | 326 | 25497 | 18861 | 2782 | 2 | 0 |
| Arab. J. Chem. | 43 | 109 | 2 | 62 | 40 | 5 | 0 | 0 |
| Arab. J. Geosci. | 34 | 38 | 1 | 19 | 14 | 4 | 0 | 0 |

**Table S16.** Publication years of documents citing the documents published in the 2009 volumes of the respective journals and present in the Scopus database at the day of analysis (cf. Table S1). Self-citations by any of the authors are excluded.

| Journal Title | Documents | Total cites | 2013 | 2012 | 2011 | 2010 | 2009 | 2008 |
| --- | --- | --- | --- | --- | --- | --- | --- | --- |
| Int. J. Cardiol. | 852 | 5698 | 10 | 1185 | 1967 | 2072 | 463 | 1 |
| J. Appl. Polym. Sci. | 1821 | 6074 | 175 | 2354 | 2129 | 1285 | 131 | 0 |
| J. Therm. Anal. Calorim. | 589 | 2046 | 26 | 744 | 701 | 525 | 50 | 0 |
| J. Radioanal. Nucl. Chem. | 458 | 823 | 12 | 266 | 314 | 207 | 24 | 0 |
| Int. J. Adv. Manuf. Technol. | 706 | 2707 | 48 | 1047 | 978 | 566 | 68 | 0 |
| Optik | 182 | 224 | 4 | 77 | 81 | 53 | 9 | 0 |
| Appl. Microbiol. Biotechnol. | 431 | 3806 | 41 | 1369 | 1329 | 908 | 159 | 0 |
| Environ. Monit. Assess. | 528 | 2233 | 28 | 834 | 784 | 499 | 88 | 0 |
| Bull. Exp. Biol. Med. | 433 | 184 | 3 | 77 | 66 | 37 | 1 | 0 |
| Oncogene | 428 | 7663 | 49 | 2459 | 2607 | 2133 | 415 | 0 |
| PLoS ONE | 4561 | 51177 | 221 | 17711 | 18883 | 12649 | 1713 | 0 |
| Arab. J. Chem. | 17 | 30 | 3 | 9 | 13 | 5 | 0 | 0 |
| Arab. J. Geosci. | N/A | N/A | N/A | N/A | N/A | N/A | N/A | N/A |

**Table S17.** Total number of citations (first three columns) and number of citations excluding self-citations by the journal (last three columns) citing any documents published in the indicated journals and included in the Scopus database at the day of analysis (cf. Table S1). The Scopus database was searched for any documents published in the indicated journals in the respective years. Citations to documents in press are included in these data as well.

| Journal Title | 2012 | 2011 | 2010 | 2012 | 2011 | 2010 |
| --- | --- | --- | --- | --- | --- | --- |
| Int. J. Cardiol. | 8740 | 12999 | 12386 | 7338 | 10657 | 10283 |
| J. Appl. Polym. Sci. | 30354 | 39437 | 36437 | 24894 | 34826 | 31690 |
| J. Therm. Anal. Calorim. | 4929 | 7055 | 6587 | 3712 | 4902 | 4233 |
| J. Radioanal. Nucl. Chem. | 4545 | 6797 | 4664 | 3112 | 4428 | 3519 |
| Int. J. Adv. Manuf. Technol. | 5669 | 8323 | 7254 | 4753 | 6843 | 5845 |
| Optik | 1019 | 1375 | 1173 | 836 | 1197 | 1024 |
| Appl. Microbiol. Biotechnol. | 16870 | 23311 | 20402 | 15880 | 21816 | 18808 |
| Environ. Monit. Assess. | 5257 | 7033 | 5592 | 4733 | 6451 | 5018 |
| Bull. Exp. Biol. Med. | 692 | 1025 | 974 | 679 | 999 | 948 |
| Oncogene | 42658 | 61595 | 60593 | 41796 | 60408 | 58963 |
| PLoS ONE | 80515 | 78246 | 43100 | 70821 | 70659 | 40558 |
| Arab. J. Chem. | 118 | 76 | 12 | 114 | 75 | 12 |
| Arab. J. Geosci. | 79 | 53 | 12 | 79 | 45 | 12 |

**Table S18.** Source web sites, dates of analyses, number of in press documents according to journals’ web sites, and dates of on-line publication of newest and oldest documents remaining in press at the analysis date. For journals published by Springer Verlag, the old version of SpringerLink was used since it allowed easier evaluation of the age of published in press documents when compared to its updated version. As a disadvantage, the old version of SpringerLink was not updated during the month preceding the analyses (as indicated in the Analysis date column). Data from these analyses were used for calculation of mean age of in press documents in each of the respective journals.

| Journal Title | Analysis date | Number of in press documents found | Newest in press document | Oldest in press document |
| --- | --- | --- | --- | --- |
| Source web site |  |  |  |  |
| Int. J. Cardiol. | 20.12.2012 | 1182 | 14.12.2012 | 2.6.2011 |
| http://www.sciencedirect.com/science/journal/01675273 | |  |  |  |
| J. Appl. Polym. Sci. | 20.12.2012 | 806 | 19.12.2012 | 17.5.2012 |
| http://onlinelibrary.wiley.com/journal/10.1002/(ISSN)1097-4628 | | |  |  |
| J. Therm. Anal. Calorim. | 21.12.2012 / 27.11.2012 | 481 | 27.11.2012 | 19.9.2005 |
| http://www.springerlink.com/content/1388-6150 | |  |  |  |
| J. Radioanal. Nucl. Chem. | 22.12.2012 / 27.11.2012 | 515 | 25.11.2012 | 24.1.2008 |
| http://www.springerlink.com/content/0236-5731/preprint/ | |  |  |  |
| Int. J. Adv. Manuf. Technol. | 22.12.2012 / 27.11.2012 | 631 | 27.11.2012 | 22.2.2005 |
| http://www.springerlink.com/content/0268-3768/ | |  |  |  |
| Optik | 22.12.2012 | 662 | 20.12.2012 | 18.4.2006 |
| http://www.sciencedirect.com/science/journal/aip/00304026 | |  |  |  |
| Appl. Microbiol. Biotechnol. | 22.12.2012 / 27.11.2012 | 377 | 27.11.2012 | 19.2.2004 |
| http://www.springerlink.com/content/0175-7598/ | |  |  |  |
| Environ. Monit. Assess. | 22.12.2012 / 27.11.2012 | 466 | 27.11.2012 | 13.6.2006 |
| http://www.springerlink.com/content/0167-6369/ | |  |  |  |
| Bull. Exp. Biol. Med. |  | 44 | 25.11.2012 | 25.11.2012 |
| http://www.springerlink.com/content/0007-4888/ | |  |  |  |
| Oncogene | 22.12.2012 | 533 | 17.12.2012 | 6.2.2012 |
| http://www.nature.com/onc/journal/vaop/ncurrent/index.html | |  |  |  |
| PLoS ONE | 23.12.2012 | 0 | N/A | N/A |
| http://www.plosone.org/ |  |  |  |  |
| Arab. J. Chem. | 23.12.2012 | 599 | 22.12.2012 | 1.10.2010 |
| http://www.sciencedirect.com/science/journal/aip/18785352 | |  |  |  |
| Arab. J. Geosci. | 23.12.2012 / 27.11.2012 | 436 | 27.11.2012 | 22.3.2011 |
| http://www.springerlink.com/content/121126/ | |  |  |  |
